# An update on ophthalmological perspectives in oculodermal melanocytosis (Nevus of Ota)

**DOI:** 10.1007/s00417-022-05743-1

**Published:** 2022-07-19

**Authors:** Solmaz Abdolrahimzadeh, Damiano Maria Pugi, Priscilla Manni, Clemente Maria Iodice, Federico Di Tizio, Flavia Persechino, Gianluca Scuderi

**Affiliations:** 1 Ophthalmology Unit, Mental Health, Neurosciences, and Sense Organs (NESMOS) Department, Faculty of Medicine and Psychology, University of Rome Sapienza, Rome, Italy; 2 St. Andrea Hospital, Via di Grottarossa 1035/1039, 00189 Rome, Italy; 3 Multidisciplinary Department of Medical Surgical and Dental Sciences, Eye Clinic, University of Campania Luigi Vanvitelli, Naples, Italy; 4 Department of Clinical and Molecular Medicine, Sapienza University of Rome, Rome, Italy

**Keywords:** Oculodermal melanocytosis, Nevus of Ota, Glaucoma, Uveal melanoma, Multimodal imaging

## Abstract

**Purpose:**

To provide a review of the literature on oculodermal melanocytosis (ODM) with a focus on the diagnostic and therapeutic implications of multimodal imaging techniques in the management of ophthalmic complications.

**Methods:**

The authors carried out a literature search on PubMed, Medline, and Scopus of English language articles published on ODM through August 2021. This review presents traditional and novel diagnostic methods in the diagnosis and follow-up of patients with particular emphasis on addressing the role of imaging in the management of the ophthalmic complications of the condition towards improving current practice patterns.

**Results:**

ODM is a rare, prevalently unilateral, congenital condition that presents with brown or blue/gray flat asymptomatic lesions of the skin, mucosae, episclera/sclera, and uvea localized within the territory of distribution of the ophthalmic and mandibular branches of the trigeminal nerve. Glaucoma and predisposition to uveal melanoma are the main ophthalmic complications. Diagnosis and management are through comprehensive opthalmological examination and traditional imaging methods such as ultrasonography and fluorescein/indocyanine green angiography as pigmentation of the fundus can conceal subtle retinal and choroidal alterations. Anterior segment optical coherence tomography and ultrasound biomicroscopy are used to evaluate the anterior segment and the ciliary body in the presence of glaucoma or melanoma of the anterior uveal tract. Fundus autofluorescence and retinal pigment epithelium (RPE) alterations are of aid in the differential diagnosis between choroidal nevi and melanoma. Enhanced depth imaging spectral domain optical coherence tomography offers outstanding in vivo evaluation of the dimensions and details of tumors or nevi and surrounding choroidal tissues and small choroidal melanomas may show distortions of the retinal and sub-retinal profile, presence of intra and sub-retinal fluid, abnormalities of the RPE, and compression of the choriocapillaris.

**Conclusions:**

Novel multimodal imaging techniques are significant in the diagnosis and management of the ophthalmic complications of ODM. Fundus autofluorescence and enhanced depth spectral domain optical coherence tomography have adjunctive value in the detection of early-stage melanoma and differential diagnosis between nevi and melanoma. Awareness of current and emerging imaging techniques can propagate improved standardized definition and assessment of the complications of ODM.


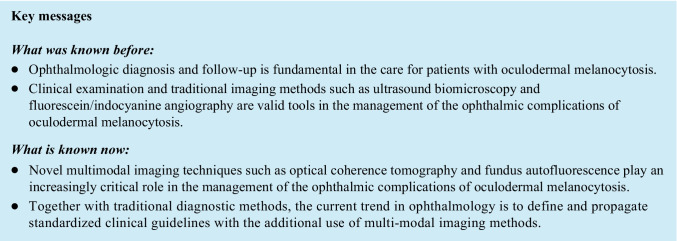


## Introduction

Oculodermal melanocytosis (ODM) or nevus of Ota is an asymptomatic, flat melanocytic lesion of the skin, and mucosae characterized by brown or blue-gray coloration, oval-shape, and poorly defined contours that can involve the episclera, sclera, and uvea of the eye [1]. It is localized within the territory of distribution of the ophthalmic and mandibular branches of the trigeminal nerve causing frequent esthetic issues. Glaucoma is one of the main ophthalmic complications and, although the melanocytosis has a benign nature, patients are predisposed to the development of uveal, orbital, and brain melanoma [2, 3]. Pigmentation of the fundus can often mask small changes [4]; thus, multimodal imaging is paramount in the detection of subtle retinal and choroidal alterations to diagnose melanoma in the early stages. This improves patient survival especially since uveal melanoma in ODM carries twice the risk of metastasis compared to uveal melanoma in eyes without ODM [5]. The present review is an analysis of the literature regarding the diagnosis, follow-up, and management of the ocular complications of ODM with updates on novel imaging methods.

## General features and etiopathogenesis

**Fig. 1:**
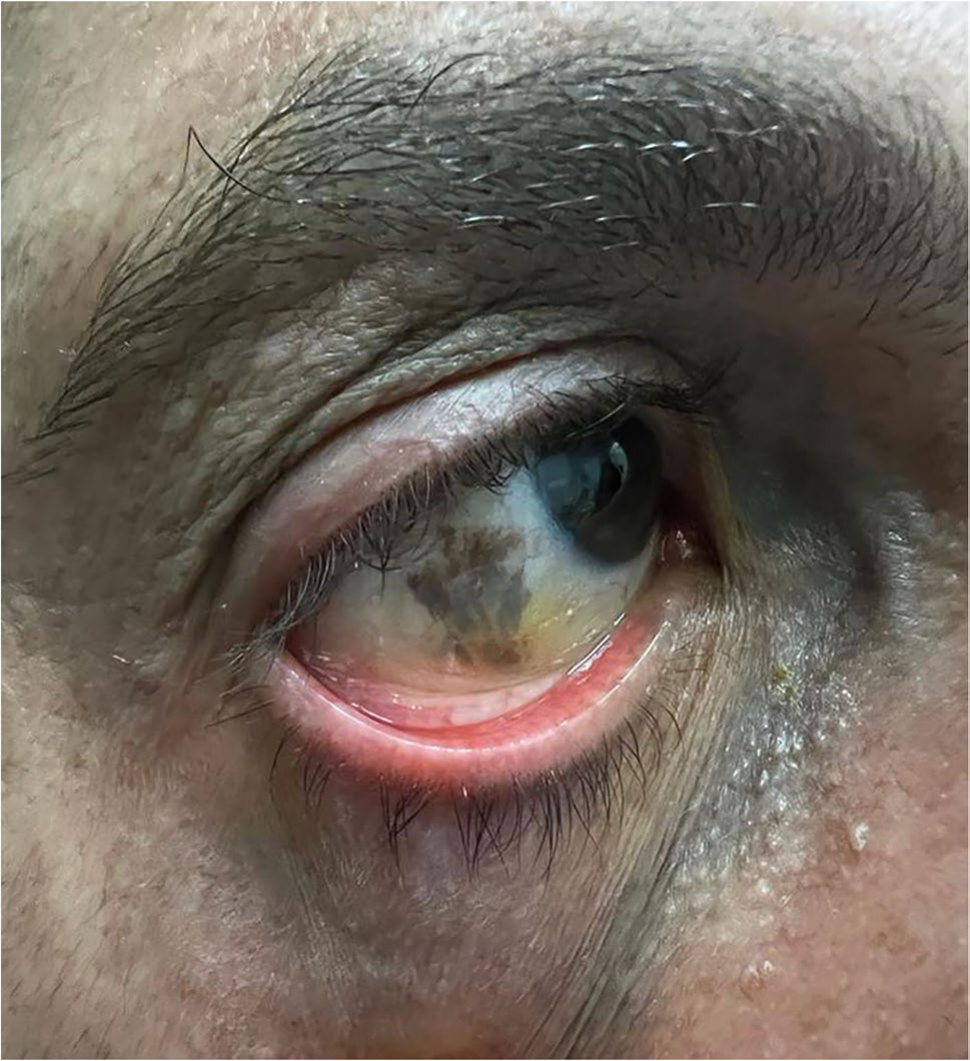
Cutaneous involvement in patient with unilateral right oculodermal melanocytosis. Evident periocular cutaneous gray-blue pigmentation that extends from the eyebrow to beneath the lower eyelid

ODM is a congenital, non-hereditary nevus, although the degree of pigmentation may increase with adolescence, adulthood, aging, and pregnancy [6]. The lesion may present a wide range of coloration due to the accumulation of melanocyte cells in a stratified manner in the dermis [7]. ODM occurs in just one eye in 90% of cases [8]. In a third of cases, only periocular skin is affected, while additional involvement of ocular tissues is reported in 66% [9] (Fig. 1). ODM does not have a propensity to recede over time; however, pigmentation may vary especially during the premenopausal period [7]. The incidence in Asians is 1–2 per 1000, whereas Caucasians are less frequently involved. There is notable difference in gender manifestation with women being five times more afflicted and this is possibly related to hormonal stimulation [10, 11]. The etiopathogenesis is linked to an alteration of the dorso-lateral migratory pathway and melanoblasts from the neural crest between the second and the eighth gestation week of embryogenesis [12].

## Classification

ODM was first described by Ota and Tanino in 1939, who proposed a classification based on the extension of the cutaneous manifestations: (1) minimum involvement with four subtypes (orbitary, zygomatic, frontal, and wing of nose), (2) medium involvement (upper and lower eyelid, periocular region, cheekbone, temple, wing, and root of nose), (3) severe involvement (type 2 plus parietal and temporal regions of the scalp, forehead, eyebrow, and nose), (4) bilateral involvement (found in about 10% of cases) [13]. Hirayama and Suzuki proposed a classification based on histopathological features: superficial dominant, diffuse, deep dominant, and deep [14]. Chan et al. proposed differentiation based on response to laser treatment: ODM without periorbital involvement, no other birthmark or extracutaneous involvement; ODM with periorbital involvement, no other birthmark or extracutaneous involvement; ODM with other birthmark but without extracutaneous involvement; ODM with extracutaneous involvement [15]. Huang et al. proposed a classification based on the innervation area of the trigeminal nerve branches: type I, II, and III involve pigmentation macules of the first, second, and third trigeminal nerve branches, respectively; type IV denotes bilateral lesion and pigmentation macules of the cheeks; type V is associated with other complications [16]. Vishnevskia-Dai et al. proposed a new classification based on ocular involvement (conjunctiva/sclera/iris/choroid) and the extent of ocular pigmentation by number of quadrants [17].

## Ocular involvement

### Episclera and sclera

**Fig. 2:**
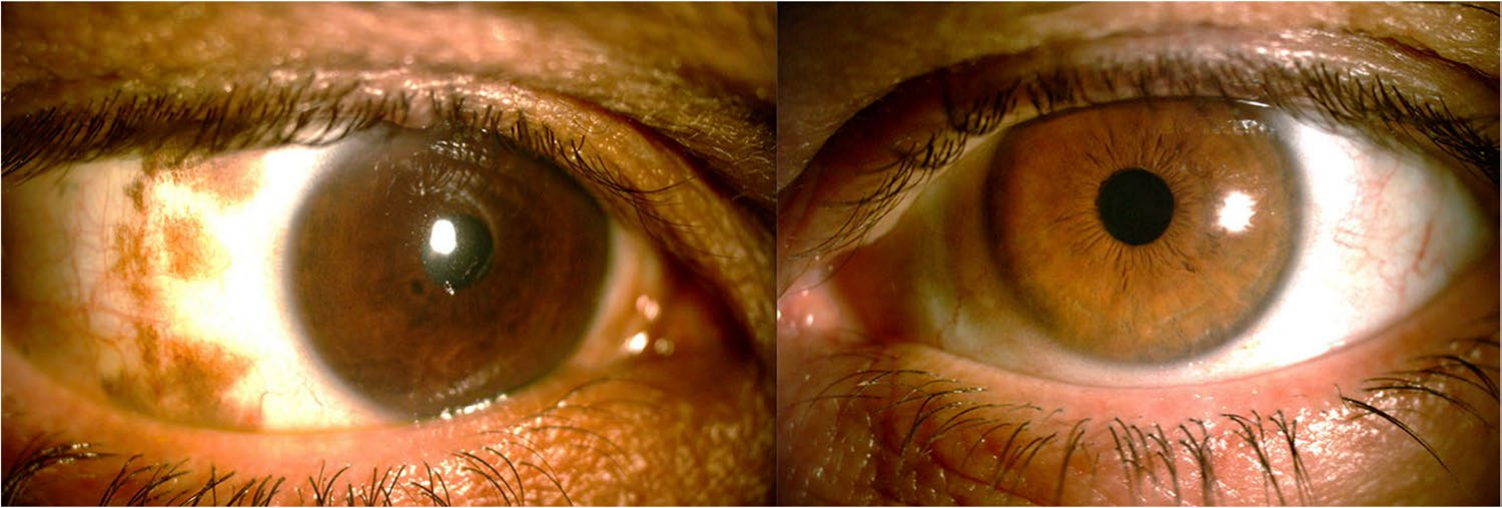
Slit lamp image of the anterior segment in a patient with unilateral right oculodermal melanocytosis. Episcleral and scleral pigmentation in the right eye is clearly visible

Accumulation of melanocyte cells at scleral and episcleral level stands out as the most common finding in ODM, most frequently in the superior temporal followed by the lower medial regions [18]. Other tissues potentially involved by infiltration of pigment are the conjunctiva, cornea, and anterior surface of the crystalline lens [19] (Fig. 2).

### Iris

**Fig. 3:**
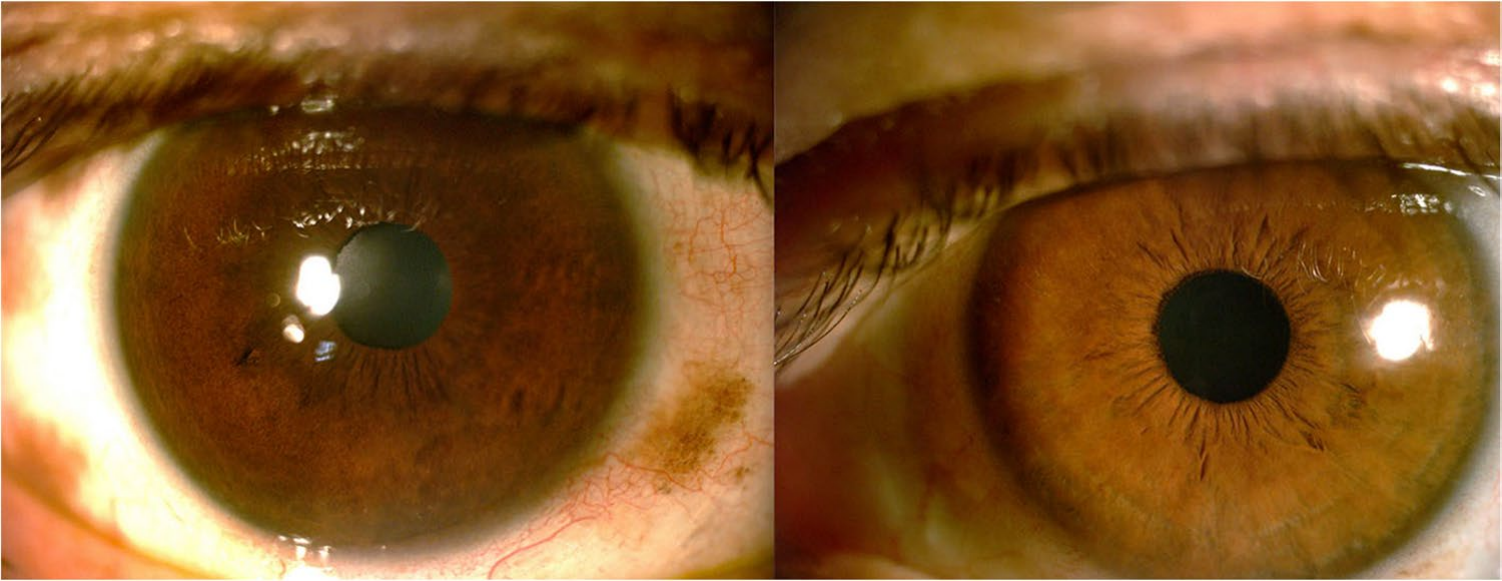
Slit lamp image of the anterior segment in a patient with unilateral right oculodermal melanocytosis. There is iris heterochromia with a darker brown coloration and less visibility of the iris crytae of the right eye. Paralimbal episcleral and scleral melanocytosis is also visible in the right eye

Patients with ODM frequently show iris heterochromia because of unilateral iris melanocytosis (Fig. 3). In rare cases, this can be associated with a velvety appearance owing to iris mammillations that are small-pigmented, homogenous nodules. Vishnevskia-Dai et al. described the loss of iris crypts due to increased thickness and reduction of flexibility of the iris stroma [17]. Iris mammillations are usually associated with increased intraocular pressure (IOP) and predisposition to intraocular melanoma [20] and should be differentiated from the Lisch nodules of neurofibromatosis type 1, which are pleomorphic in appearance, lighter in color, and bilateral [20, 21].

An iris melanoma should be suspected in the presence of an elevated iris mass especially if it shows features such as elevated intraocular pressure, iris seeding, ciliary body invasion, extraocular extension, or growth [22].

### Ciliary body

Ocular melanocytosis can lead to development of ciliary body melanoma. Evaluation of the ciliary body with UBM at regular intervals can be helpful for early detection of ciliary body melanoma in eye with ocular melanocytosis [23]. Although there are well-defined criteria for the diagnosis of small choroidal melanoma, such criteria do not exist for small melanoma of the ciliary body. A possible distinguishing factor could be lesion growth, but it has been shown that even nevi can grow slowly over long periods of time [24]. The most common technique to obtain tissue for definite diagnosis is needle biopsy for cytopathologic examination [24].

### Fundus oculi

**Fig. 4:**
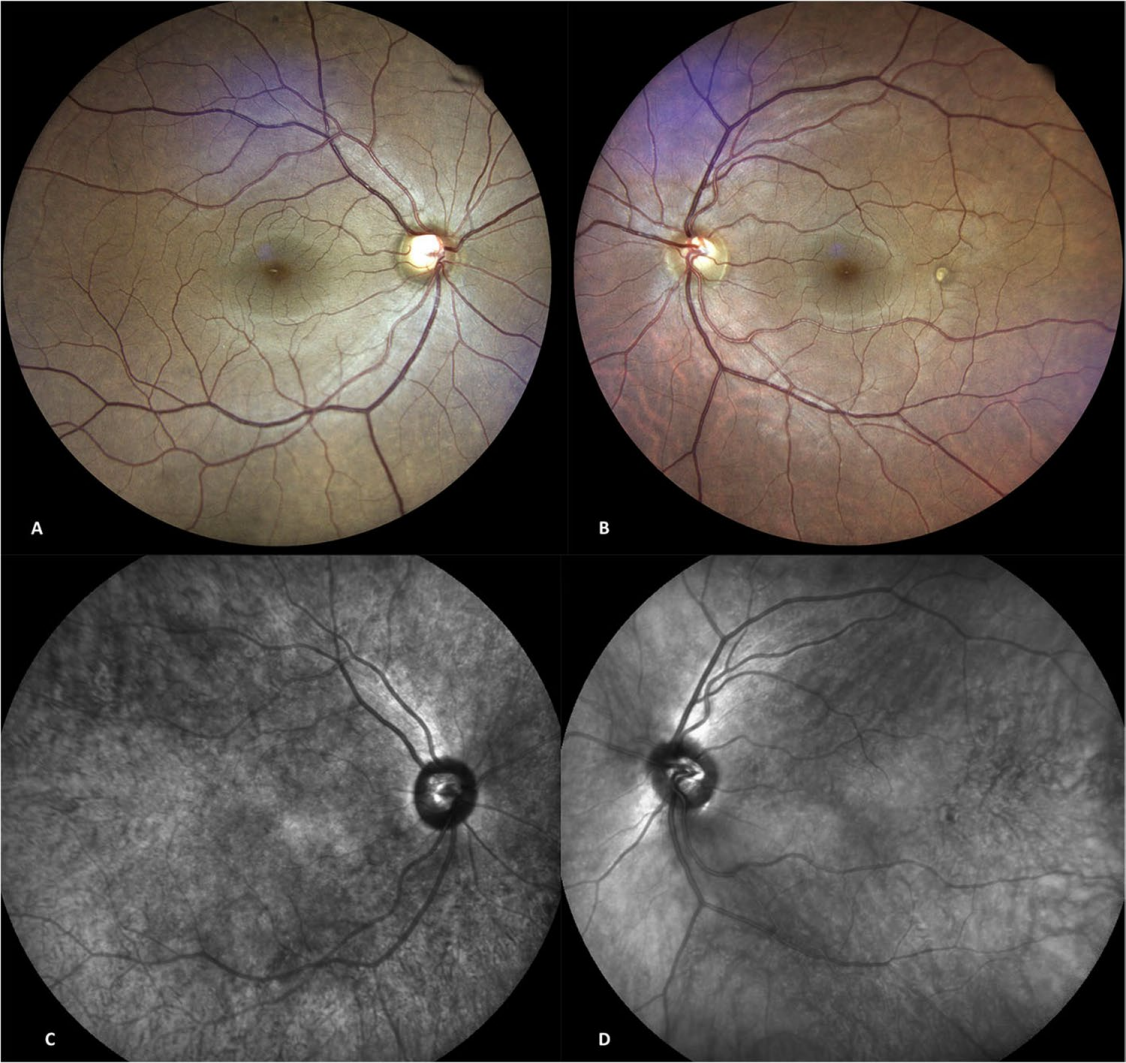
Bilateral fundus photographs and near infrared reflectance (NIR) images of the posterior pole in a patient with unilateral right oculodermal melanocytosis. There is a diffuse darker coloration and rare mottled focal hyperpigmention of the right (**A**) with respect to the left (**B**) fundus. The fundus tessellation is more evident in the left, unaffected, eye. There is asymmetric optic nerve excavation between the two eyes. The NIR image of the right eye (**C**) is notably darker with a mottled appearance and reduced visibility of choroidal vessels with respect to the left eye (**D**)

The fundus oculi generally shows a darker color in the affected with respect to the fellow eye (Fig. 4). The cause is excessive choroidal pigmentation that can occasionally give rise to pigmentary mottling and dots. Fine et al. illustrated the case of an optic disk hemangioblastoma, which was presumably embryologically related to ODM due to the neuroectodermal nature of both lesions [25]. Kumar et al. reported on a patient showing a singular association between ODM and primary retinitis pigmentosa [26].

## Ocular complications

### Glaucoma

Open-angle glaucoma may develop because of the abnormalities of the irido-corneal angle or the accumulation of pigment in the trabecular meshwork and Schlemm’s canal resulting in aqueous humor outflow obstruction [27, 28]. Glaucoma is normally ipsilateral to the pigmentation and occurs in 10% of patients with ODM and periodic follow-up is highly recommended [29]. Glaucoma can also develop when ODM is associated with other neuro-oculocutaneous diseases, such as the Sturge-Weber syndrome, Klippel-Trenáunay syndrome, and phakomatosis pigmentovascularis [30–34].

### Melanoma

ODM increases the risk of developing melanoma of the periorbital skin, uveal tract, orbit, and central nervous system. Uveal melanoma develops more frequently in the white population but cases in Asians have also been described. It is estimated that one out of 400 white patients with pre-existing ODM develops uveal melanoma during their lifetime [35] and ODM is up to 35 times more common in patients with uveal melanoma [36]. ODM is also a risk factor for the development of atypical presentations of uveal melanoma, such as bilateral and multifocal melanoma [23, 37]. A somatic activating mutation of the guanine nucleotide-binding protein (GNAQ) gene or a biallelic mutation of the BAP1 gene was reported in 85% of uveal melanomas and 15% of ODM [38]. GNAQ and BAP1 mutations were reported to predispose to metastatic tumor progression [39]. Pan et al. described three cases of choroidal melanoma in Chinese patients with ODM with genome studies showing a potential correlation between the mutation of FAM111B and DSC2 genes and tumor development [40].

Shields et al. examined 7872 patients with uveal melanoma and evaluated the weight of the simultaneous presence of ODM on the rate of metastasis and death. They reported patients with uveal melanoma associated with ODM have double the risk for metastasis compared with those with no melanocytosis and suggested follow-up every 6 months in order to enable early detection of any possible melanoma [5].

Cases of cutaneous melanoma in ODM have been reported in the literature. Patterson et al. described a papulous lesion developing from a macular pigmentary region that was diagnosed as a cutaneous melanoma [41] and Baroody et al. analyzed a melanoma of the deep dermis [42]. Rarely, melanoma can infiltrate the orbital area [43] or affect meningeal nervous tissue [44].

## Associated conditions

The pigmentary cells in ODM can involve retrobulbar fat, periostium, extra ocular muscles, palate, buccal mucosa, tympanic membrane, and meningeal tissue [45]. Several cases of meningeal melanocytoma have been described [46]. Luo et al. outlined a rare case of association with vitiligo [47], while Alvarez-Cuesta et al. depicted a case of ODM with ipsilateral deafness [48]. Furthermore, ODM may overlap with other neuro-oculo-cutaneous conditions and the phakomatoses [32, 34].

## Diagnosis

Diagnosis requires a multidisciplinary approach including dermatological and ophthalmological evaluation. The dermatological manifestations can create psychological issues for the patient due to esthetic implications, while the ophthalmological involvement can lead to severe eye complications.

### Dermatological evaluation

Dermatological evaluation is primarily carried out with noninvasive dermatoscopy, although diagnosis is not always straightforward. Reflectance confocal microscopy is a noninvasive, in vivo, real-time histological imaging technique that enables to obtain high-resolution images of the cutaneous lesions showing pigmentary spindle-shaped cells scattered within the collagen of the dermis [49]. This virtual biopsy enables to distinguish between the high refractivity or homogeneity of dendritic cells of ODM compared to the pleomorphism of shape and size of melanocyte cells [50]. Cutaneous biopsy can provide more definite diagnosis and hematoxylin–eosin staining enables adequate assessment of melanocytic cells, as they appear bipolar, dendritic, and fragmented within packages of collagen with an irregular distribution [51].

### Ophthalmological evaluation of the anterior segment

The approach to the anterior segment is through the use of the slit lamp, tonometry, gonioscopy, ultrasound biomicroscopy, and anterior segment optical coherence tomography (ASOCT).

#### Slit lamp, gonioscopy, and tonometry

Slit lamp examination enables to observe the site and extension of pigmentation, heterochromia of the iris, and iris mammillations. Gonioscopy can reveal the possible presence of pigment in the irido-corneal angle. Although a specific pattern of angle pigment distribution and accumulation in ODM patients is not described in the literature, its role in the pathogenesis of glaucoma is well recognized [52]. Both iris melanocytosis and pigment dispersion syndrome can be associated with pigmentary glaucoma caused by deposition of pigment in the trabecular meshwork. Whereas glaucoma associated with pigment dispersion syndrome is usually bilateral, glaucoma due to iris melanocytosis is usually unilateral and limited to the involved eye [53, 54].

#### Ultrasound biomicroscopy

Ultrasound biomicroscopy (UBM) is an immersion technique that uses high-frequency ultrasound to obtain high-resolution in vivo images of the anterior segment, with acquisition of images at a depth of 4–5 mm and a resolution of 25–50 µm. This technique has numerous applications including assessment of the anterior chamber angle, evaluation of alterations and tumors of the iris and ciliary body, and measurement of anterior and posterior chamber dimensions. Velazquez-Martin et al., in a retrospective observational case series, used UBM to analyze ciliary body alterations in patients with unilateral ODM using the unaffected fellow eyes for comparison and found increased thickness and reflectivity of the ciliary body due to both augmented melanocyte cells and increased pigmentation [23]. The presence of pigmentary lesions involving the iris in the context of ODM should lead to further assessment with UBM. The ciliary body is evaluated by UBM to check for the presence of a tumor and to measure the dimensions of the tumor [55]. In glaucoma associated with ODM, angle amplitude abnormalities, and the increase in size of the iris, the ciliary body due to pigment accumulation can be detected [56].

#### Anterior segment optical coherence tomography

ASOCT is an imaging method with an axial resolution of 15 µm and a wavelength of 1300 nm that enables the acquisition of images of the anterior segment. In ODM, ASOCT allows the assessment of the conjunctiva, sclera, cornea, and irido-corneal angle. Although the method provides good imaging of the frontal surface of pigmentation, it has limited ability to penetrate deeper for the assessment of the posterior margins of pigmented lesions and the ciliary body [57]. In the study of glaucoma associated with ODM, ASOCT may help by creating cross-sectional images of the irido-corneal angle, iris, and anterior chamber in order to obtain quantitative parameters [58].

### Ophthalmological evaluation of the posterior segment

The evaluation of the fundus oculi is necessary in order to periodically monitor choroidal pigmentation, excavation of the optic disk, and possible development of melanomas. Evaluation involves indirect ophthalmoscopy or biomicroscopy and multimodal imaging with fundus autofluorescence (FAF), fluorescein angiography (FA), indocyanine green angiography (ICGA), spectral domain OCT (SDOCT), OCT angiography, and ultrasonography.

#### Fundus autofluorescence

FAF is a noninvasive method that enables to evaluate the retinal pigment epithelium (RPE). It is based on the emission of light by the granules of lipofuscin. Cennamo et al. studied fundus autofluorescence aspects in 100 choroidal nevi and 65 choroidal melanomas in the differential diagnosis of choroidal melanoma versus nevi. They found normal background fundus fluorescence and overlying choroidal nevi in forty eyes. In sixty eyes, hypoautofluorescence of choroidal nevi was associated with RPE alterations. In contrast, they found plaque-like hyperautofluorescence on the surface of choroidal melanoma in twenty-six cases corresponding to pigment augmentation and fibrous metaplasia [59].

#### Fluorescein angiography and indocyanine green angiography

FA is a method used for the evaluation of the retinal circulation by administering an intravenous bolus of fluorescein sodium salt. Normally, the details of the choroidal circulation is not visible when the RPE is intact. ICGA, carried out with a water-soluble dye with high protein binding capacity that does not pass through the fenestrations of the choriocapillaris, is used in the study of the choroidal circulation owing to the ability of infrared radiation emitted by indocyanine to penetrate through the normal ocular pigments. On FA, choroidal melanoma shows areas of blotched hyper-fluorescence, late staining linked to the presence of a mass and areas of diffuse hyper-fluorescence due to the sub-retinal localization of overlying fluid. In contrast, a choroidal nevus typically remains hypo-fluorescent without overlying RPE leaks. ICGA shows an area of hyper/hypo-fluorescence depending on the size of the tumor, whereas nevi tend to demonstrate hypo-fluorescence if compared to the surrounding choroid [60, 61].

#### Spectral domain optical coherence tomography

Spectral domain OCT (SDOCT) is a technique that uses a light source with a wavelength of around 850 nm to visualize all retinal layers with great resolution and sensitivity. The enhanced depth imaging mode (EDI-SDOCT) enables evaluation of the choroid up to the scleral-choroidal interface and is fundamental in detecting early changes associated with development of melanoma.

**Fig. 5:**
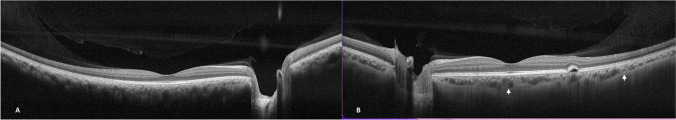
Spectral domain optical coherence tomography in a patient with unilateral right oculodermal melanocytosis. The horizontal line scan shows similar retinal structure and thickness in both eyes. In the right eye, the choroidal scleral junction is not visible due to increased choroidal thickness (**A**), whereas this is clearly visible in the unaffected left eye (**B**) (incidental presence of small temporal macular pigment epithelial detachment in the left eye)

Pellegrini et al. used EDI-SDOCT in 15 patients with unilateral choroidal melanocytosis and compared the features with the un-involved fellow eye. They reported defects in the myoid zone and the ellipsoid junctions in involved eyes. Choroidal features were a “smooth anterior choroidal contour, and thinned or compressed choriocapillaris, thinned or thickened medium vessels, and thinned large vessels.” Furthermore, they found that mean subfoveal choroidal thickness was increased by 23% and choroidal perivascular tissue was increased by 51% in the study eye. The authors speculated that melanocytosis increases choroidal thickness owing to increased tissue cellularity since they found an increased ratio between perivascular stromal thickness and entire choroidal thickness, measuring 66% in the affected and 54% in the fellow eye, respectively [4] (Fig. 5).

Possible evidence of uveal melanoma shown with SDOCT is distortions of the retinal and sub-retinal profile, presence of intra and sub-retinal fluid, and abnormalities of the RPE such as atrophy, accumulations of lipofuscin, compression of the choriocapillaris, and presence of dome-shaped choroidal masses [62].

EDI-SDOCT enables measurement of the height and diameter of posterior choroidal lesions that are too small to be identified using ultrasonography [63]; furthermore, the latter may overestimate height of lesions. Daitch et al. described a patient with heterochromia and unilateral ODM where a small subclinical choroidal melanoma was diagnosed presenting as a choroidal mass with overlying sub-retinal fluid and shaggy photoreceptors on EDI-SDOCT [64].

Shields et al. reviewed a series of 37 eyes with small choroidal melanoma and reported that EDI-SDOCT offers an outstanding in vivo evaluation of tumor dimensions and cross-sectional details of both the tumor and surrounding choroidal tissue. Compared to choroidal nevi, small choroidal melanomas more frequently showed increased tumor thickness, sub-retinal lipofuscin deposition, sub-retinal fluid, and RPE atrophy. The authors reported that small choroidal melanomas tended to show shaggy (irregular, elongated, and presumed swollen) photoreceptors, disappearance of the external limiting membrane and junction between inner and outer segments, inner plexiform layer and ganglion cell layer irregularities, and intraretinal edema [65].

SDOCT enables to analyze the neuronal and axonal components of the retina and optic disk with parameters such as retinal fiber layer/ganglion cell layer/inner plexiform layer thickness, ganglion cell complex (GCC) thickness, optic nerve head (ONH), and peripapillary retinal nerve fiber layer (RNFL) thickness. These parameters may present changes in glaucoma associated with ODM [66].

#### Optical coherence tomography angiography

Optical coherence tomography angiography (OCTA) is a noninvasive high-resolution imaging technique that enables to visualize the retinal and choroidal circulation. Quantitative and qualitative evaluation of the superficial and deep retinal vascular plexuses, the choriocapillaris flow area, and the foveal avascular zone (FAZ) can be assessed. Thus, the microvascularity of the macular area in uveal melanomas can be studied. The FAZ may or may not be enlarged in both the superficial and deep vascular plexuses. In choroidal nevus, OCTA shows a hyporeflective mass that does not significantly alter choroidal vascularization and the EPR-Bruch’s membrane complex [67].

#### Ultrasonography

Ultrasonography represents the cardinal imaging method when opacities of the lens and the cornea are present or in cases where a thorough evaluation of intraocular tumors is needed. Both mono-dimensional (A-scan) or bi-dimensional (B-scan) modes should be integrated. The use of posterior segment ultrasonography in melanoma is indicated in the assessment of tumor mass size and in the follow-up after radiation therapy. Differential diagnosis is with choroidal metastasis, choroidal hemangioma, and nevi.

A-scans for melanoma show low to medium internal reflectivity, sound attenuation pattern (angle k), and rapid spontaneous echo signals that point out blood flow in tumor vessels [68, 69]. On B-scans, choroidal melanomas can be dome, mushroom, or collar-button shaped in cases where the tumor penetrates the Bruch’s membrane or presents in a diffuse fashion. Furthermore, B-scan distinctive traits are acoustic quiet zone, shadowing behind the mass, uniform consistency with no relevant acoustic interfaces, low to medium reflectivity, heartbeat-synchronized vascularity, and choroidal excavation [70].

Nevi on A-scan ultrasonography show high internal reflectivity. Differential diagnosis of nevi from melanomas through ultrasonography is based on the evaluation of both mass thickness and base diameter. Masses under 1 mm of thickness, diagnosed as nevi, do not tend to convert into melanomas; however, the cutoff for potential melanomas is above 2 mm in thickness, 7 mm of base diameter, and evident signs of growth [71]. Choroidal hemangioma presents high internal reflectivity on A-scan ultrasonography, in contrast to low to medium internal reflectivity in choroidal melanomas [72].

## Treatment

Patients with ODM pursue esthetic treatment solutions due to emotional stress issues of physical deformity. The best results are when skin surgery is performed within the early stages of disease. Cryotherapy, dermabrasion, and skin grafting have not been effective and currently laser therapy represents the best approach [73]. Q-switched (QS) laser considerably reduces side effects such as post-inflammatory hyperpigmentation or hypopigmentation by providing short pulses of energy [74]. Amaki et al. found that QS Nd:YAG laser is an effective and safe measure for scleral treatment in the selective laser trabeculoplasty mode on 13 patients. Reported side effects were edema and mild subconjunctival hemorrhages which resolved after a few days and 2 weeks, respectively [75].

Bum-Joo Cho et al. described scleral allograft overlay in eight eyes of 7 patients with scleral nevus of Ota and reported good esthetic outcome and minimal complications [76]. Tae-im Kim et al. used flipped scleral flap surgery to remove ODM pigmentation as superficial sclera is more pigmented than the deeper sections; however, this surgery is longer, more complex, and complete removal of all abnormal pigmentation is rarely achieved [77]. Jung et al. reported good results with superficial sclerectomy. This method reduces operating times and the scleral bed is smoother [78]. However, the set back is possible over-weakening of the sclera due to estimation of the depth of sclerectomy during surgery. Mularoni et al. reported a case where ASOCT images were acquired prior to sclerectomy in order to better evaluate the depth of surgery that was then performed with a precalibrated diamond blade [79].

### Glaucoma treatment

Management of glaucoma in ODM requires a thorough clinical assessment to classify the type of glaucoma and make a well-balanced decision for the most appropriate medical and/or surgical treatment [29]. Among the first-line IOP lowering drugs are prostaglandin analogues and alpha-agonists. Beta-blockers and carbonic anhydrase inhibitors are second-line treatment due to their systemic adverse effects and lack of effectiveness, respectively. In open-angle glaucoma, laser trabeculoplasty (SLT) can be used as first-choice treatment or in association with medical therapy [80]. Chaiwat et al. described acutely elevated lOP ipsilateral to ODM together with anterior uveitis in five patients. All patients positively responded to treatment with anti-glaucoma drugs and topical steroids and surgery was not required [81]. The detection of progression of damage shown by visual field defects or SDOCT alterations of the optic nerve, together with the failure to reach target pressure indicates the need to evaluate filtering or valve surgery [80, 82].

### Uveal melanoma treatment

The treatment of uveal melanoma aims to preserve vision and to prevent metastatic diffusion. Almost 50% of patients with uveal melanomas are shown to develop metastasis 15 years after diagnosis [83]. The principal options for local small size melanoma are transpupillary thermotherapy and radiotherapy subdivided in brachytherapy and proton beam radiotherapy [84]. Ultrasound, used intraoperatively, highly improves the precision of tumor localization for brachytherapy and proton beam radiotherapy [85].

ODM has the potential to undergo malignant transformation to melanoma, where it commonly presents in one of two locations: retro-orbital (within the intraconal space) or in the uveal tract [86]. The treatment of melanoma associated with ODM is similar to melanoma not associated with ODM [36]. There are a few case reports or case series in the literature specifically on ODM associated with uveal melanoma. Qiong et al. described three cases of uveal melanoma in ODM; two patients were surgically treated with vitrectomy and local tumor resection and one patient was given transpupillary thermotherapy [87]. Al-Sadhan et al. reported a case of uveal melanoma associated with ODM. Diagnosis of melanoma was confirmed after histological examination of the enucleated eye [88]. Rao et al. and Fallon et al. reported enucleation in multifocal choroidal melanomas in ODM [89, 90]. Daitch et al. described a case of melanoma diagnosed with EDI-SDOCT and treated with plaque radiotherapy. In these cases, the risk of metastasis is greater and early treatment is recommended [64].

## Conclusions

ODM poses esthetic challenges because of visible facial pigmentary alterations but also increases the risk of glaucoma and uveal melanoma in patients. Ophthalmologists have a decisive role in the early diagnosis and management of these ocular complications. Traditional imaging and novel imaging methods with EDI-SDOCT and OCTA are valid and promising methods to facilitate the early diagnosis and management of potentially sight and life-threatening complications.

## Data Availability

Not applicable.
